# Efficient Massive MIMO Detection for M-QAM Symbols

**DOI:** 10.3390/e25030391

**Published:** 2023-02-21

**Authors:** Zhi Quan, Jiyu Luo, Hailong Zhang, Li Jiang

**Affiliations:** School of Electrical and Information Engineering, Zhengzhou University, Zhengzhou 450001, China

**Keywords:** box-constrained dichotomous coordinate descent, massive MIMO, negative diagonal loading regularization, signal detection, quadrature amplitude modulation

## Abstract

Massive multiple-input multiple-output (MIMO) systems significantly outperform small-scale MIMO systems in terms of data rate, making them an enabling technology for next-generation wireless systems. However, the increased number of antennas increases the computational difficulty of data detection, necessitating more efficient detection techniques. This paper presents a detector based on joint deregularized and box-constrained dichotomous coordinate descent (BOXDCD) with iterations for rectangular m-ary quadrature amplitude modulation (M-QAM) symbols. Deregularization maximized the energy of the solution. With the box-constraint, the deregularization forces the solution to be close to the rectangular boundary set. The numerical results demonstrate that the proposed detector achieves a considerable performance gain compared to existing detection algorithms. The performance advantage increases with the system size and signal-to-noise ratio.

## 1. Introduction

Massive multiple-input multiple-output (MIMO) systems are well known as essential enablers for the future of wireless communication. An antenna array with many antennas enables time-frequency resources to be shared by all user terminals at the same time. Massive MIMO is better than traditional MIMO in many ways, such as making links more reliable and using less energy [1]. Quadrature amplitude modulation (QAM) provides extra benefits for data transmission. Unfortunately, interference from multiple antennas becomes much worse as the size of the system increases, making uplink data detection a tough problem. As a result, to estimate a signal, advanced signal processing techniques are needed.

The MIMO data detection problem can be constructed as a linear system of equations. The maximum likelihood (ML) technique theoretically provides the optimal detection performance, but its computational cost is prohibitively high for massive MIMO systems. Therefore, suboptimal detectors with low complexity and strong detection performance are required in practical applications [2]. Sphere decoder (SD) can achieve near-ML performance while requiring less computational complexity than ML detectors. Its computational complexity, however, is inconsistent with the large-scale MIMO problem [3]. Belief propagation (BP) detectors provide the soft output in an iterative process via a factor graph and suffer less local minimum problems than other algorithms. However, BP-based algorithms show severe performance loss as the ratio of the number of receive antennas to the number of transmit antennas approaches one [4,5]. Moreover, as the number of antennas increases, the complexity of a BP detector itself becomes a burden when the number of antennas becomes larger. Zero forcing (ZF) is simple to implement but provides poor detection performance. However, the linear system of equations has a channel matrix of ill-determined rank, and Tikhonov regularization is a prominent method for solving this problem. A ZF detector with Tikhonov regularization, referred to as a minimum mean-square error (MMSE) detector, obtains a better performance–complexity tradeoff than nonlinear detection algorithms. In addition, linear MIMO receivers including ZF and MMSE detectors need to perform channel correlation inversion.

Matrix inversion requires a complexity of $O(N^{3})$ (where *N* is the system size), which is too high for large system implementation. To avoid the exact matrix inversion needed by linear detection in a MIMO uplink, various sophisticated techniques have been proposed, which can be generically divided into three categories: approximate matrix inversion methods, decomposition methods, and iterative methods [6]. Neumann series expansion is applied to approximate matrix inversion using a series of matrix–vector multiplications, which is simple to implement in hardware but converges slowly [7]. Newton iteration [8] and Chebyshev iteration [9] have been successively proposed to accelerate convergence. However, when the number of iterations exceeds one, their computational complexity exceeds that of exact matrix inversion [10]. The complexity of decomposition methods such as Cholesky decomposition, Gaussian elimination, and QR decomposition (QRD) is $O(N^{3})$. Iterative methods such as steepest descent (SD) and conjugate gradient (CG) achieve rapid convergence, but each iteration requires $O(N^{2})$ mathematical operations. Methods of coordinate descent (CD), such as Jacobi [11], Gauss‒Seidel [12], and successive overrelaxation (SOR), have slower convergence but have only O(N) operations per iteration. The computational complexity of these iterative techniques is proportional to the number of iterations executed. Only if the ratio of base station (BS) antennas to user antennas ($\rho =\frac{K_{r}}{K_{t}}$) is large enough can satisfactory performance and a reasonable level of complexity be reached. However, a sufficient number of receive antennas may not be available in a practical system due to the constraints of antenna size, cost, and power consumption. Systems with ρ close to one can achieve better spectral efficiency when attaining large $K_{t}$ and $K_{r}$. However, the performance of the linear detectors degrades dramatically. Excessive dimensionality reduction for the sake of computational reduction results information loss, and substantial performance degradation is unavoidable due to interference, particularly when $K_{t}$ is of the same order as $K_{r}$. Furthermore, multiplication and division operations are required for these iterative methods. Numerical instability may occur during division operations [13]. These operations are all thought to be difficult to implement in hardware [14].

The dichotomous coordinate descent (DCD) algorithm is based on the coordinate descent method. It solves the normal equation by using variable step sizes with a power of two. With this algorithm, a linear system of equations can be solved with high computational efficiency. The algorithm is easy to implement in hardware because it does not require multiplications and divisions [15]. DCD-based multiuser detectors have demonstrated a detection accuracy close to the single-user bound for binary phase shift keying (BPSK) symbols [16,17]. Better detection performance is achieved using box-constrained optimization [18]. In [19], a box-constrained DCD detector and its hardware architecture were proposed for complex-valued detection. Nevertheless, this architecture design was based on a serial structure and can only be suitable for small-scale systems. In [20], a parallel FPGA architecture for a box-constrained MIMO detector based on DCD iteration is proposed for MIMO detection with tens/hundreds of antennas. The complexity of these DCD-like detection methods mainly depends on the number of antennas and the number of successful update iterations $N_{u}$. With a high $N_{u}$, DCD-based detectors show good performance for large-scale systems. However, the complexity burden requirement will become severe as the system size increases. To further reduce complexity without sacrificing detection capability, designing suboptimal detection algorithms with low complexity and high performance is still needed to construct practical MIMO systems.

To address this problem, a massive MIMO detector based on an iterated box-constrained DCD (BOXDCD) with a negative diagonal loading regularization is proposed. The idea is that the output of the BOXDCD algorithm with negative diagonal loading regularization provides a rough estimation of the m-ary quadrature amplitude modulation (M-QAM) symbols for massive MIMO systems and is taken as the initial input to the BOXDCD algorithm when updating the received signal vector. This procedure is repeated iteratively, achieving a significant performance improvement and an overall complexity reduction compared to the MMSE detector. Moreover, the proposed detector achieves much higher performance than the BOXDCD detector with the same $N_{u}$. We provide a complexity–performance tradeoff for the proposed efficient massive MIMO detector compared with suboptimal MIMO detectors under a fully loaded system and show that the proposed method outperforms existing suboptimal detectors.

The remainder of the paper is organized as follows: Section 2 describes the system model for the MIMO transmission system and briefly overviews the MMSE detector and iterated MMSE detector. The proposed detector based on an iterated box-constrained DCD algorithm with negative diagonal loading is presented in Section 3. Numerical simulations are shown in Section 4. Section 5 concludes the work.

Mathematical notations: matrices and column vectors are denoted by bold uppercase and lowercase letters, respectively. The elements of the matrix and vector are denoted as $R_{i,j}$ and $r_{j}$, respectively. The *j*-th column of R is denoted as $R_{:,j}$. The real and imaginary parts of a complex number are denoted by ℜ(·) and ℑ(·), respectively. The inverse and conjugate transpose are represented by ${\left(\cdot \right)}^{-1}$ and ${\left(\cdot \right)}^{H}$, respectively. I stands for an identity matrix.

## 2. Preliminaries

We begin by introducing the system model used in MIMO detection. The MMSE and box-constrained DCD algorithms used to solve the linear model are then discussed.

### 2.1. Problem Formulation

We consider an uplink massive MIMO system in which the BS is equipped with $K_{r}$ receiving antennas and $K_{t}$ transmitting antennas. The received signal $z\in C^{K_{r}}$ at the BS can be expressed as

(1) z=Hs+w,

where $H\in C^{K_{r}\times K_{t}}$ denotes a complex channel matrix whose elements are independent and identically distributed (i.i.d.) zero mean Gaussian random numbers, s is a $K_{t}\times 1$ transmitted complex vector from a $2^{2x}$(*x* is an integer)-order quadrature amplitude modulation (QAM) constellation A (e.g., 4QAM, 16QAM), and w denotes a $K_{r}\times 1$ i.i.d. complex additive white Gaussian noise (AWGN) vector with zero-mean and covariance matrix $\sigma^{2}I$.

The ML detector solves (1) with the best detection performance. Assuming H is known at the receiver, the ML detector is equivalent to solving the problem below:

(2) $$\begin{array}{ccc} {\hat{s}}_{\mathrm{ML}} & = & \arg\min_{s\in A^{K_{t}}}||z-{\mathrm{Hs}||}^{2}\\ & = & \arg\min_{s\in A^{K_{t}}}\left\{s^{H}Rs-2ℜ\left\{y^{H}s\right\}\right\}, \end{array}$$

where $R=H^{H}H$ and $y=H^{H}z$. Unfortunately, the fact that the ML signal detector is NP-hard (nondeterministic polynomial time) prevents the use of the method that provides a perfect solution (2).

### 2.2. MMSE Detector

To reduce the complexity, the MMSE detector relaxes the constellation constraints $s\in A^{K_{t}}$ to $s\in C^{K_{t}}$ and adds a regularization term to the Equation (2) as follows:

(3) $${\hat{s}}_{\mathrm{mmse}}=\arg\min_{s\in C^{K_{t}}}\left\{{∥y-\mathrm{Rs}∥}^{2}+{\mu ||Ls||}^{2}\right\},$$

with regularization operator *L* and regularization parameter μ>0. By setting L=I and $\mu =\sigma^{2}$, the solution to (3) is given by

(4) $${\hat{s}}_{\mathrm{mmse}}={\left(R+\sigma^{2}I\right)}^{-1}y.$$

The iterative Tikhonov regularization technique helps to obtain a more accurate solution. By applying this technique to Equation (3), we can obtain a sequence of constrained optimizations.

(5) $$s^{\left(v\right)}=\arg\min_{s\in C^{K_{t}}}∥\left(y+\sigma^{2}s^{\left(v-1\right)}\right){-\mathrm{Rs}∥}^{2}+\sigma^{2}{\left||Is|\right|}^{2}$$

The solution of the *v*th iteration to Equation (5) can be considered as an unconstrained solution to the following equation:

(6) $$s_{\mathrm{iterated}\;\mathrm{mmse}}^{\left(v\right)}={\left(R+\sigma^{2}I\right)}^{-1}\left(y+\sigma^{2}s^{\left(v-1\right)}\right)$$

Although the MMSE approach has been demonstrated to be efficient for small-scale MIMO systems (e.g., at most eight antennas in LTE-A), it requires matrix inverse operation. In massive MIMO systems with hundreds of antennas, the matrix inverse operation has a prohibitively large complexity of $O\left(K_{t}^{3}\right)$ (and $O(\left(V-1\right)K_{t}^{3})$ for iterated MMSE) [13].

## 3. Detector Based on Iterated BOXDCD with Negative Diagonal Loading Regularization

### 3.1. Box-Constrained Minimization

A promising detection method referred to as box-constrained optimization constrains the estimated symbol vector to be within a closed convex set and has been shown to outperform linear detection methods [18]. An additional tightening of the solution can further improve the performance. The box-constrained method relaxes the constraint $s\in A^{K_{r}}$ in (2) to the convex polytope $s\in U_{A}$, which is defined as follows:

(7) $$U_{A}=\left\{s_{R}+js_{I}:s_{R},s_{I}\in \left[-\xi ,+\xi \right]\right\},$$

where $s_{R}^{2},s_{I}^{2}\leq E_{\mathrm{MQAM}}/2$, and ξ is the radius of the tightest box around the square constellation. For 4QAM, ξ=1. For higher-order QAM constellations, such as 16QAM or 64QAM, ξ=3 or 7.

When applying the box-constrained method to solve the ML problem in a MIMO system, problem (2) is reformulated as

(8) $$\hat{s}=\arg\min_{s\in U_{A}}\left\{s^{H}Rs-2ℜ\left\{y^{H}s\right\}\right\}.$$

The condition number of the channel matrix can be altered by changing the relationship between $K_{t}$ and $K_{r}$. Typically, when $K_{t}$ is closer to $K_{r}$, the matrix condition number is larger. Matrices with high condition numbers may decrease the robustness of the detection. Regularization with negative diagonal loading maximizes the solution’s energy. When regularization is used with box constraints, the solution is forced to be close to the boundary. For example, the QAM constellation signal input s satisfies $s^{T}s=\kappa$ and hence solves the inequality $(\kappa -s^{H}s)\geq 0$. To further tighten the solution, we can rearrange Equation (8) as

(9) $$\begin{array}{ccc} \hat{s} & = & \arg\min_{s\in U_{A}}\left\{s^{H}Rs-2ℜ\left\{y^{H}s\right\}+\mu \left(\kappa -s^{H}s\right)\right\}\\ & = & \arg\min_{s\in U_{A}}\left\{s^{H}\left(R-\mu I\right)s-2ℜ\left\{y^{H}s\right\}\right\} \end{array}$$

The matrix (R−μI) implies that regularization with negative diagonal loading is used. The regularization parameter μ is chosen to be the same as in the MMSE detector. The DCD algorithm has shown excellent efficiency in implementing box-constrained detectors [21].

### 3.2. Combination of Box-Constrained Minimization with the DCD Algorithm

The box-constrained DCD (BOXDCD) algorithm, shown in Algorithm 1, is used to solve a $K_{t}\times K_{t}$ complex-valued linear system ($y=\overset{´}{R}\cdot s$). The matrix $\overset{´}{R}$ and vectors y, χ and s are complex-valued. The matrix $\overset{´}{R}$ and vector y are known, and the solution s is unknown. The residual vector χ is represented by $\chi =y-\overset{´}{R}s$, where s is initialized to $s_{0}$. The vector χ element contains both real and imaginary parts, which are processed sequentially. The variable *f* determines whether a component is the real part (f=1) or the imaginary part (f=j). To apply the BOXDCD algorithm to solve a linear complex-valued system $y=\overset{´}{R}s$, we can reformulate it to an equivalent real-valued system by the following rule: $\bar{y}=\left[ℜ\{y\right\},ℑ\{y{\}]}^{T}\in R^{2K_{t}}$, $\bar{s}={\left[ℜ\left\{s\right\},ℑ\left\{s\right\}\right]}^{T}\in R^{2K_{t}}$ and $\bar{R}=\left(\begin{array}{cc} ℜ\{\overset{´}{R}\} & -ℑ\{\overset{´}{R}\}\\ ℑ\{\overset{´}{R}\} & ℜ\{\overset{´}{R}\} \end{array}\right)\in R^{2K_{t}\times 2K_{t}}$.
**Algorithm 1:** BOXDCD algorithm ($K_{t}\times K_{t}$ system).       **Step**   **Operation**+
Input: $M_{b},N_{u}$, $s_{0},y,\overset{´}{R}$
Output: s, χ
*initialization*
$s=s_{0}$, $\chi =y-\overset{´}{R}s$, λ=ξ, counter=0, f=1
for $\;\delta =1,\ldots ,M_{b}$1    λ=λ/22    g=0                            ⤏ pass
   for $\;n=1,\ldots ,K_{t}$        ⤏ iteration3       if f=1, do $\chi_{temp}=ℜ\left(\chi_{n}\right)$, else $\chi_{temp}=ℑ\left(\chi_{n}\right)$1
      if $|\chi_{temp}|>\left(\lambda /2\right){\overset{´}{R}}_{n,n}$ do4         $s_{nt}=s_{n}+\mathrm{sign}\left(\chi_{temp}\right)f\lambda$15          if $ℜ(s_{nt})\leq \xi$ and $ℑ(s_{nt})\leq \xi$, then6          $s_{n}=s_{nt}$
         $\chi =\chi -\mathrm{sign}\left(\chi_{temp}\right)f\lambda {\overset{´}{R}}_{:,n}$$K_{t}$7          counter=counter+1, g = 18          if $counter=N_{u}$, algorithm exits9       if f=1, do f=j, goto step 3; else f=1
10   if g=1, goto step 2


The BOXDCD algorithm updates the solution s elements in the order $n=1,2,\cdots ,K_{t}$. The iteration counter is initialized to zero when the binary variable *g* is set to 1, which means that the current iteration is “successful”. For “successful" iterations, the vectors χ, $s_{n}$ and index *n* are updated. $M_{b}$ denotes the number of bits of s within the range [−ξ,ξ]. The initial value of the step size λ is defined by the parameter ξ. In each step size, the algorithm goes through all the elements of s and repeats the process until $|\chi_{temp}|\leq \left(\lambda /2\right){\overset{´}{R}}_{n,n}$. The step size λ is reduced at step 1 and chosen as a power of two, which can be implemented as shift by bits. In each iteration, updating $s_{n}$ at step 4 requires one addition and a bit shift. At step 6, updating χ requires $K_{t}$ additions and bit-shift operations. The complexity load of the algorithm mainly depends on the number of successful update iterations. To limit the algorithm’s complexity, the maximum number of successful update iterations $N_{u}$ is established. In this paper, when the box-constrained DCD algorithm is used, we denote it as BOXDCD($s_{0},\overset{´}{R},y,N_{u},M_{b}$). The complexity of the BOXDCD algorithm for solving a system of equations varies depending on the system size, successful update iterations $N_{u}$ and the conditioning of the channel matrix. The algorithm’s worst-case complexity is bounded by $K_{t}\left(2N_{u}+M_{b}-1\right)+N_{u}$ shift-accumulation operations with given $N_{u}$ and $M_{b}$. In practice, however, there should be several successful iterations in each pass. The average complexity of the BOXDCD algorithm is close to $2K_{t}N_{u}$. For dealing with a $K_{t}\times K_{t}$ system, the real part and the imaginary part are processed sequentially. The whole average computational complexity of the BOXDCD is approximately $4K_{t}N_{u}$.

An iterative method can obtain a more accurate solution to Equation (9). Here, applying an iteration with negative diagonal loading regularization yields the following solution:

(10) $${\hat{s}}^{\left(v\right)}=\arg\min_{s\in U_{A}}\left\{s^{H}Rs-2ℜ{\left(y+\mu {\tilde{s}}^{\left(v-1\right)}\right)}^{H}s-\mu s^{H}s\right\}$$

where ${\tilde{s}}^{\left(-1\right)}=0$, ${\tilde{s}}^{\left(v-1\right)}={\hat{s}}^{\left(v-1\right)}$. The solution to (10) can be regarded as the solution to the equation

(11) $$\begin{array}{ccc} \left(R-\mu I\right){\hat{s}}^{\left(v\right)} & = & y+\mu {\tilde{s}}^{\left(v-1\right)}\\ ⟹\overset{´}{R}{\hat{s}}^{\left(v\right)} & = & y^{\left(v\right)}. \end{array}$$

In each iteration, Equation (11) can be solved using the BOXDCD algorithm. Consequently, we obtain the iterated BOXDCD with a negative diagonal loading (INL-BOXDCD) detector for M-QAM symbols, which is summarized in Algorithm 2 and the individual optimization steps are given below.

**Step 1:**  The BOXDCD algorithm is applied to solve equation $y=\overset{´}{R}s$. s is initialized as $s_{0}$ and $\overset{´}{R}=R-\mu I$, where regularization parameter $\mu =\eta \sigma^{2}$.
**Step 2:**  The soft output ${\tilde{s}}_{0}$ obtained from step 1 is demodulated and mapped into the M-QAM constellation to obtain ${\hat{s}}^{\left(0\right)}$.
**Step 3:**  For each iteration, the vector y is updated as $y^{\left(v\right)}=y+\mu {\hat{s}}^{\left(v-1\right)}$. Then, the BOXDCD is applied with ${\hat{s}}^{\left(v-1\right)}$ and the updated y to obtain the solution vector ${\hat{s}}^{\left(v\right)}$.
**Step 4:**  ${\bar{s}}^{\left(V\right)}$ is mapped into the M-QAM constellation to obtain $\hat{s}$.

For a given $N_{u}$, the INL-BOXDCD detector has an average computational complexity of $4K_{t}N_{u}\left(1+V\right)$ additions for a $K_{t}\times K_{t}$ MIMO system.

**Algorithm 2:** Proposed INL-BOXDCD detector.   **Step**
**Operation**
Input: $s_{0}$, R, y, ξ, $N_{u}$, $M_{b}$, μOutput: $\hat{s}$

Initialization

$s=s_{0}$, $\overset{´}{R}=R-\mu I$1Use $\mathrm{BOXDCD}(s,\overset{´}{R},y,N_{u},M_{b})$ to solve $y=\overset{´}{R}s$ to obtain ${\tilde{s}}_{0}$.2Map ${\tilde{s}}_{0}$ into the M-QAM constellation to obtain ${\hat{s}}^{\left(0\right)}$.3

for


v=1,2,3⋯,V

   $y^{\left(v\right)}=y+\mu {\hat{s}}^{\left(v-1\right)}$   Use $\mathrm{BOXDCD}({\hat{s}}^{\left(v-1\right)},\overset{´}{R},y^{\left(v\right)},N_{u},M_{b})$ to solve $y^{\left(v\right)}=\overset{´}{R}s^{\left(v\right)}$ to obtain ${\tilde{s}}^{\left(v\right)}$.

end


for

4Map ${\tilde{s}}^{\left(V\right)}$ into the M-QAM constellation to obtain $\hat{s}$.

## 4. Simulation Results

In this section, numerical results obtained by MATLAB simulations are presented. We consider a large-system limit in which $K_{r}$ and $K_{t}$ are tens to hundreds of antennas at a proportional rate $\rho =\frac{K_{t}}{K_{r}}\leq 1$. To assess the error-rate performance for the proposed INL-BOXDCD algorithm, we perform Monte Carlo simulations of a MIMO system adopting different forms of QAM under a Rayleigh flat fading channel. The simulation trials are $10^{6}$. In this scenario, $M_{b}=15$, η=0.1. The simulation results are plotted as the bit-error-rate (BER) vs. the signal-to-Gaussian noise ratio (SNR).

**Figure 1:**
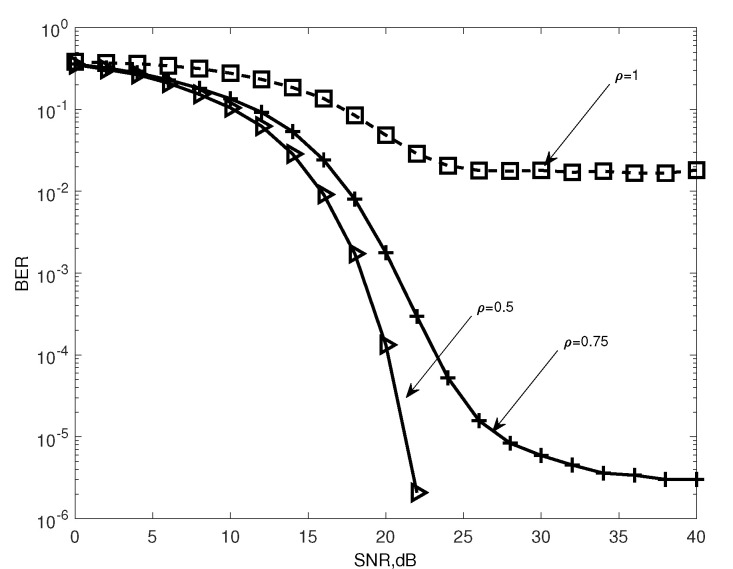
BER performance of the INL-BOXDCD as a function of SNR for different ratios ρ of receiving to transmitting antennas; V=1, $N_{u}=300$, 16QAM and $K_{t}=60$.

Figure 1 illustrates the detection performances of the INL-BOXDCD detector for 16QAM symbols for ρ=60/120,60/80 and 60/60 with V=1. We can alter the condition number of the channel correlation matrix R by changing the relationship between $K_{r}$ and $K_{t}$. When ρ=1, we address fully loaded MIMO systems, and the noise is magnified by a factor of a relatively large condition number. Under such conditions, the convergence property of INL-BOXDCD deteriorates. With the decreases in ρ, the performance of the INL-BOXDCD detector significantly increases. Increasing *V* might mitigate the negative effects of approximation error.

**Figure 2:**
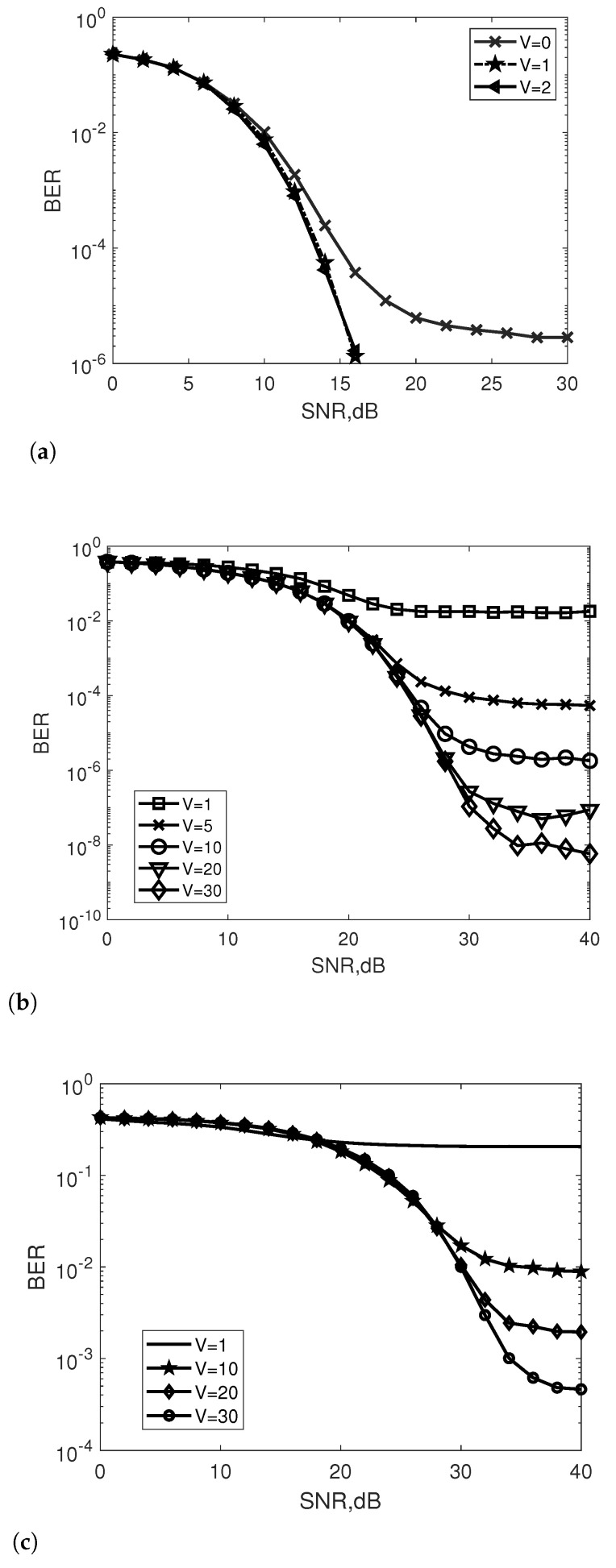
Effect of *V* on the BER performance of the INL-BOXDCD detector for a 60×60 MIMO system with different QAM constellations; (**a**) 4QAM; (**b**) 16QAM; (**c**) 64QAM.

Figure 2 depicts the effect of iterations *V* on the BER performance of the INL-BOXDCD detector under 4QAM, 16QAM and 64QAM for a 60×60 MIMO system with $N_{u}=300$. For 4QAM symbol transmission (Figure 2a), the INL-BOXDCD detector with one iteration provides a significant accuracy improvement over zero. However, after one iteration, the BER performance of the INL-BOXDCD detector can no longer be improved. The INL-BOXDCD detector for 16QAM and 64QAM signals with V=1 shows poor performance. When *V* increases to 10, 20 and 30, the BER gradually decreases. With a given $N_{u}$, Figure 2b,c show that the INL-BOXDCD exhibits a BER floor that is reduced as *V* increases.

**Figure 3:**
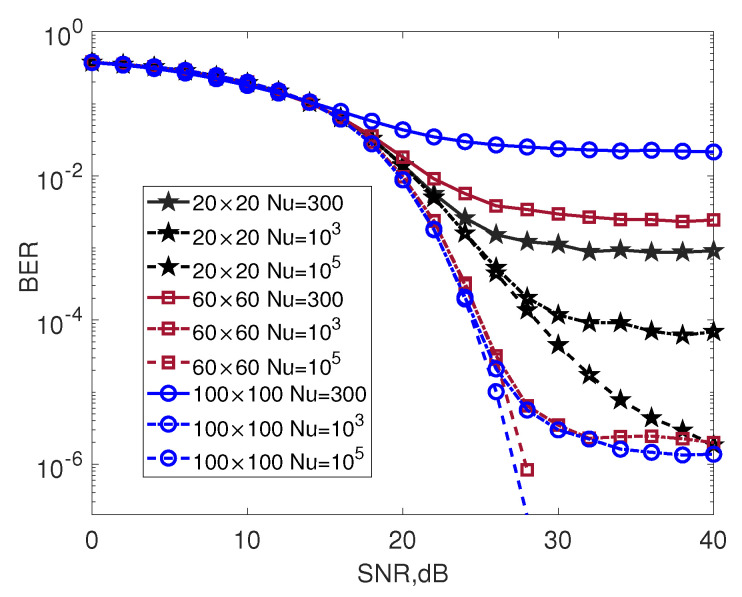
The effect of $N_{u}$ on the BER performance for different MIMO configurations; 16QAM, V=2.

Figure 3 shows the effect of $N_{u}$ on the BER performance of the INL-BOXDCD detector for 20×20, 60×60 and 100×100 MIMO systems. With a fixed $N_{u}$, the INL-BOXDCD displays a BER floor and the BER floor decreases when $N_{u}$ increases. For a small value of $N_{u}=300$, the INL-BOXDCD detector for the 20×20 MIMO system offers better performance than the 60×60 and 100×100 MIMO systems. When $N_{u}$ increases to $10^{3}$, the INL-BOXDCD detector with large-scale MIMO systems, such as the 60×60 and 100×100 systems, shows significantly better performance than the 20×20 MIMO system. When $N_{u}$ further increases to $10^{5}$, the BER performance of the INL-BOXDCD detector for the 60×60 MIMO system is slightly worse than that of the 100×100 MIMO system but significantly better than that of the 20×20 MIMO system.

**Figure 4:**
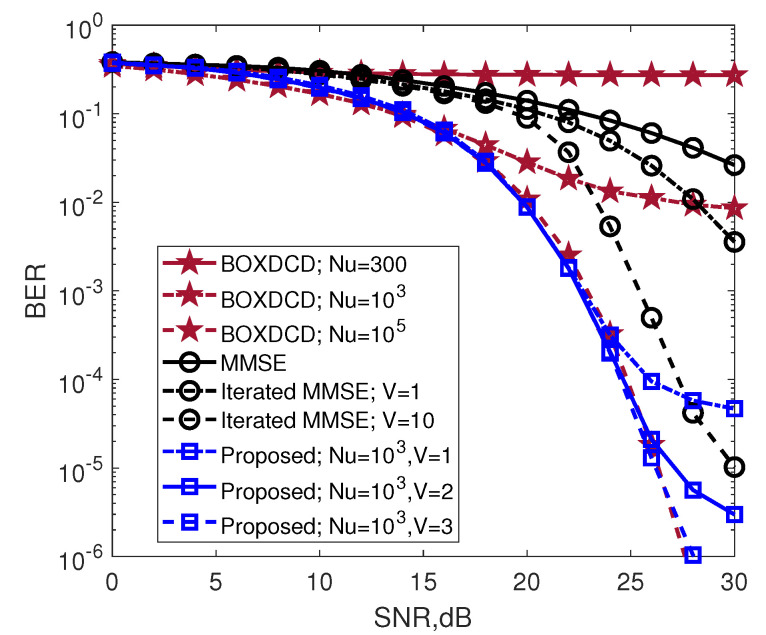
BER performance comparison among BOXDCD, MMSE and INL-BOXDCD: 16QAM, 100×100 MIMO configuration.

Figure 4 plots the BER performance of the INL-BOXDCD detector for a given SNR for a 100×100 antenna configuration. The performance of the MMSE, iterated MMSE and BOXDCD are also examined. The figure shows that the performance of the INL-BOXDCD detector improves significantly with increasing $N_{u}$ and *V*. The detection performance of the classical MMSE detector is inferior to that of the INL-BOXDCD detector. The INL-BOXDCD ($N_{u}=10^{3},V=1$) detector provides a gain of approximately 3 dB over iterated MMSE (V=10) when BER is less than $10^{-4}$. The BOXDCD detector with $N_{u}=300$ provides poor performance. With increasing $N_{u}$, the detection performance of the BOXDCD detector improves. As *V* increases from 1 to 3, the BER performance curve of the INL-BOXDCD detector with $N_{u}=10^{3}$ gradually approaches that of the BOXDCD detector with $N_{u}=10^{5}$. At BER=$1.3\times 10^{-5}$, the average complexity of the INL-BOXDCD (with $N_{u}=10^{3}$ and V=3) is $0.16\times 10^{5}K_{t}$, and the average complexity of the BOXDCD (with $N_{u}=10^{5}$) is $4\times 10^{5}K_{t}$. When $N_{u}=10^{3},V=1$, the computational complexity of the INL-BOXDCD detector is twice as high as that of the BOXDCD detector, while the INL-BOXDCD detector has a BER that is approximately 100 times lower after SNR=24 dB.

The average complexity of the proposed INL-BOXDCD algorithm for a 100×100 MIMO system is listed in Table 1 and compared with that of the MMSE, iterated MMSE (V=10), BOXDCD$\left(N_{u}=10^{5}\right)$ and INL-BOXDCD$\left(N_{u}=10^{3},V=3\right)$ when BER = $10^{-4}$. For $K_{t}=100$, the MMSE detector approximately requires ${K_{t}}^{3}=10^{6}$ complex multiplication and addition operations. The average complexity of the iterated MMSE detector with V=10 is ${K_{t}}^{3}\left(V-1\right)=9\times 10^{6}$ complex multiplications and additions. With a given $N_{u}=10^{5}$, the average computational complexity of BOXDCD is $4K_{t}N_{u}=40\times 10^{6}$ real additions. For a given $N_{u}=10^{3}$ and V=3, the average computational complexity of the INL-BOXDCD detector is $4K_{t}N_{u}\left(V+1\right)=1.6\times 10^{6}$ real additions. From Table 1 and Figure 4, we notice that the MMSE detector shows poor performance, while iterative MMSE has a better performance by increasing *V*. When BER = $5\times 10^{-5}$, the iterated MMSE requires 2 dB more than BOXDCD and INL-BOXDCD. The matrix inversion of iterated MMSE (and MMSE) requires high complexity $O({K_{t}}^{3})$, which is calculated by counting the cost of the addition and multiplication operations. BOXDCD and INL-BOXDCD require only addition operations. The results also show that the proposed algorithm, INL-BOXDCD, has a much lower computational cost compared to BOXDCD by reducing $N_{u}$, while the two algorithms achieve comparable performance.

**Figure 5:**
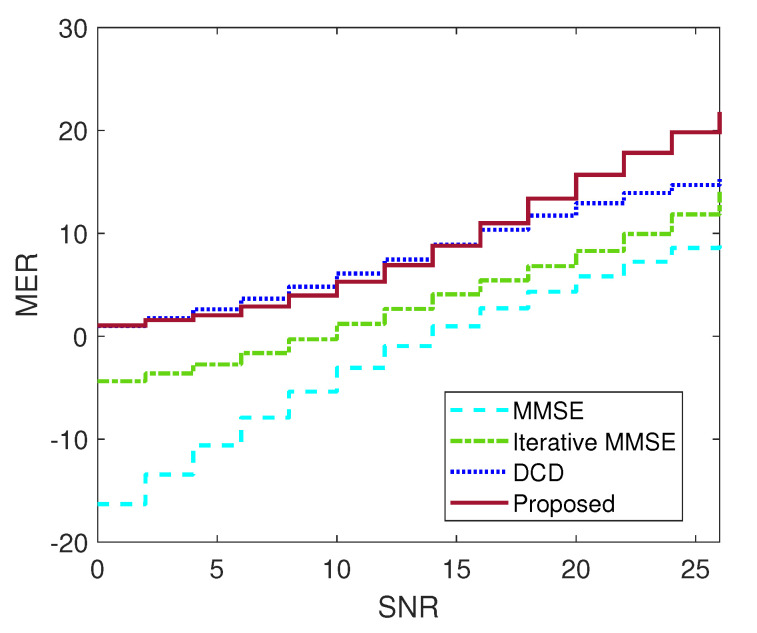
MER performance comparison among MMSE, iterated MMSE (V=1), BOXDCD ($N_{u}=10^{3}$) and INL-BOXDCD ($N_{u}=10^{3}$, V=1): 16QAM, 100×100 MIMO configuration.

The modulation error ratio (MER) performance of MMSE, iterated MMSE, BOXDCD and INL-BOXDCD over 1000 16QAM symbols in a 100×100 MIMO system are shown in Figure 5. The results show that for a large-scale system, box-constrained detectors offer better performance than MMSE-type detectors. The INL-BOXDCD shows a better performance than BOXDCD with $N_{u}=10^{3}$ after 15dB, which is consistent with the BER verifications.

### Discussion

The MMSE detectors provide a good soft decision for MIMO detection; however, they are difficult to implement in large MIMO systems. In addition, the box-constrained-based detectors can achieve significantly better performance than the MMSE detector in a large MIMO system [19]. Iterated MMSE detectors avoid the BER floor while requiring a large complexity of $\left(V-1\right)K_{t}^{3}$, which is not implemention friendly in massive MIMO systems. The INL-BOXDCD algorithm uses iterated BOXDCD algorithms with deregularization to solve normal equations. For a fixed $N_{u}$, the BOXDCD exhibits a BER floor at a high SNR. Increasing $N_{u}$ reduces the BER floor. The performance and computational complexity of INL-BOXDCD mainly depend on the number of bits $M_{b}$, updates $N_{u}$ and iterations *V*. Using lower $N_{u}$ and iterated BOXDCD algorithms, the INL-BOXDCD detector could achieve a good tradeoff between performance and complexity compared to only applying the BOXDCD detector. According to the BOXDCD algorithm FPGA design in [20], the method requires 184, 210 and 223 slices for the system sizes of 16×16, 64×64 and 128×128 in Xilinx Virtex-6 FPGA (XC6VSX475T-2FF1759). To achieve the same performance, the INL-BOXDCD algorithm can be performed by using a single BOXDCD block with less processing time. It might be an alternative way to solve a fully loaded MIMO system.

## 5. Conclusions

In this work, we consider massive MIMO detection as a quadratic optimization problem and propose a solution based on an iterated BOXDCD algorithm with negative diagonal loading regularization. Negative diagonal loading regularization maximizes the energy of the solution to the box boundary. We examine the tradeoff between the computational complexity and the performance among MMSE, iterated MMSE, BOXDCD and INL-BOXDCD and demonstrate that the proposed detector INL-BOXDCD outperforms the more advanced counterparts and provides much greater adaptivity in a fully loaded massive MIMO system. In this work, we only consider a single user case MIMO scenario; performance investigation of the proposed method in multiuser MIMO systems is left for future work.

## Figures and Tables

**Table 1:** Average complexity comparison of the detection methods.

Method	Complexity Order
MMSE	$10^{6}$ (complex multiplications and additions)
iterated MMSE	$9\times 10^{6}$ (complex multiplications and additions)
BOXDCD	$40\times 10^{6}$ (real additions)
INL-BOXDCD	$1.6\times 10^{6}$ (real additions)

## Data Availability

Not applicable.
